# Cartilage Degradation in Psoriatic Arthritis Is Associated With Increased Synovial Perfusion as Detected by Magnetic Resonance Imaging

**DOI:** 10.3389/fmed.2020.539870

**Published:** 2020-09-25

**Authors:** Daniel B. Abrar, Christoph Schleich, Anja Müller-Lutz, Miriam Frenken, K. Ludger Radke, Stefan Vordenbäumen, Matthias Schneider, Benedikt Ostendorf, Philipp Sewerin

**Affiliations:** ^1^Department of Diagnostic and Interventional Radiology, Medical Faculty, University Dusseldorf, Düsseldorf, Germany; ^2^Department and Hiller Research Unit for Rheumatology, UKD, Heinrich Heine University Düsseldorf, Düsseldorf, Germany

**Keywords:** psoriatic arthritis, magnetic resonance imaging, dGEMRIC, cartilage, compositional imaging

## Abstract

**Objective:** Even though cartilage loss is a known feature of psoriatic arthritis (PsA), research is sparse on its role in the pathogenesis of PsA and its potential use for disease detection and monitoring. Using delayed gadolinium-enhanced magnetic resonance imaging of cartilage (dGEMRIC) and dynamic contrast-enhanced MRI (DCE MRI), research has shown that early cartilage loss is strongly associated with synovial inflammation in rheumatoid arthritis (RA). The aim of this study was to determine if acute inflammation is associated with early cartilage loss in small finger joints of patients with PsA.

**Methods:** Metacarpophalangeal (MCP), proximal interphalangeal (PIP), and distal interphalangeal (DIP) joints of 17 patients with active PsA were evaluated by high-resolution 3 Tesla dGEMRIC and DCE MRI using a dedicated 16-channel hand coil. Semi-quantitative and quantitative perfusion parameters were calculated. Images were analyzed by two independent raters for dGEMRIC indices, PsA MRI scores (PsAMRIS), total cartilage thickness (TCT), and joint space width (JSW).

**Results:** We found significant negative correlations between perfusion parameters (except K_ep_) and dGEMRIC indices, with the highest value at the MCP joints (K_Trans_: τ = −0.54, *p* = 0.01; K_ep_: τ = −0.02, *p* = 0.90; IAUC: τ = −0.51, *p* = 0.015; Initial Slope: τ = −0.54, *p* = 0.01; Peak: τ = −0.67, *p* = 0.002). Heterogeneous correlations were detected between perfusion parameters and both, total PsAMRIS and PsAMRIS synovitis sub-scores. No significant correlation was seen between any perfusion parameter and JSW and/or TCT.

**Conclusion:** As examined by DCE MRI and dGEMRIC, there is a potential association between early cartilage loss and acute synovial inflammation in small finger joints of PsA patients.

## Introduction

Psoriatic arthritis (PsA) is a chronic autoimmune disease that ultimately leads to joint destruction and functional disability (1). As in rheumatoid arthritis (RA), early diagnosis and treatment are pivotal for a better clinical outcome (2, 3). Therefore, treat-to-target (T2T) strategies have been introduced for the treatment of PsA (4, 5). Even though magnetic resonance imaging is not yet included in the classification criteria for PsA (CASPAR), it becomes increasingly important for the early detection and monitoring of disease-related joint changes (6–8). In 2009, the Outcome Measures in Rheumatology Clinical Trials (OMERACT) working group introduced a semi-quantitative PsA MRI score (PsAMRIS) that evaluates metacarpophalangeal (MCP), proximal (PIP), and distal interphalangeal joints concerning the osteodestructive (bone erosion), osteoproliferative (bone proliferation), and acute inflammatory (synovitis, flexor tenosynovitis, periarticular inflammation) features of PsA (9). Several studies have shown that the degree of synovial contrast enhancement in arthritic joints can be quantified by dynamic contrast-enhanced MRI (DCE MRI), and hence, have found a strong correlation between the synovitis sub-score of PsAMRIS and RA MRI score (RAMRIS) and DCE MRI parameters (10–12). Furthermore, elevated synovial perfusion assessed by DCE MRI reflects histological findings of acute synovitis (13). Even though cartilage damage is a known feature of PsA, research is sparse on its value in the pathogenesis and the disease course (14). That is why it is not included in the PsAMRIS as opposed to its RA equivalent (15). Several studies using delayed gadolinium-enhanced MRI of cartilage (dGEMRIC) have shown that early cartilage loss in RA is associated with the severity of synovitis (10, 16). dGEMRIC is a histologically validated and robust method that depicts proteoglycan loss in cartilage by measurement of fixed-charge density (17, 18). Proteoglycans have negatively charged side chains that allow for the inversely proportional penetration of similarly negatively charged contrast agent molecules (e.g., gadolinium) following intravenous administration. Consequently, proteoglycan depletion leads to an accumulation of gadolinium ions in degenerated cartilage.

However, the placement of region of interests in small joints is difficult using conventional MRI or with high-resolution MRI surface coils. We, therefore, used a 16-channel high-resolution hand coil to allow for an improved evaluation of smaller joints.

Herein, we set out to evaluate if there was any association between acute inflammation and early cartilage loss in small finger joints of patients with PsA.

## Methods

### Study Population

Seventeen patients with PsA (mean age 53.7 ± 11.6; minimum/maximum 26/72 years, male/female 9/8) fulfilling the CASPAR criteria, mean disease duration 4 ± 3.6 years, and suffering from peripheral joint involvement of at least two MCP joints and dactylitis of at least one finger were prospectively recruited for the “Analysis of the DActylic Melange” (ADAM) research initiative. All patients had failed methotrexate (MTX) monotherapy and were escalated to etanercept therapy after a baseline MRI scan. Patient recruitment took place in the Department of Rheumatology from 06/ 2015 to 01/ 2017. The same study population has been included in a different study. However, this study has been published as a pre-print only (19).

The study was approved by the local ethics committee (study number: 4962R, “Analysis of the Dactylitic Melange (ADAM): Defining the morphological components of dactylitis in psoriatic arthritis and their responsiveness to etanercept therapy). Written and informed consent was obtained from all patients before initiation of the study. The Disease Activity Score 28 (DAS 28) was 2.42 ± 0.72 (range 1.8–4.3, median 2.2). C-reactive protein (CRP) levels were 0.87 ± 1.35 mg/dl (range 0.1–5.8 mg/dl, median 0.3 mg/dl).

### MR Imaging

A 3T MRI scanner (Magentom Skyra, Siemens Healthineers, Erlangen, Germany) and a dedicated 16-channel hand coil (3T Tim Coil [receive only], Siemens Healthineers, Erlangen, Germany) was used for all patients. Patients were imaged in the prone position with their arm extended overhead (“superman position” with palm facing down).

The imaging protocol included coronal T1-weighted turbo spin echo (TSE) sequences before and after intravenous injection of an ionic gadolinium-based contrast agent (Gd-DOTA^−^ [Dotarem, Guerbet Villepinte, France] in double dose, 0.4 mmol/kg bodyweight). The intravenous injection was carried out by an injection pump followed by a saline chaser. Also, non-contrast enhanced, fat-saturated T2-weighted/short tau inversion recovery (STIR) as well as post-contrast fat-saturated T1-weighted sequences in at least two different orthogonal planes were obtained.

Compositional MRI using the dGEMRIC technique of the MCP, PIP, and DIP joints 2–5 was performed 40 min after intravenous contrast-agent administration. To this end, we used a flip-angle three-dimensional gradient-echo (GE) imaging (FLASH) sequence with two excitation flip angles (5° and 26°) as in previously published studies of our institute (17, 20, 21). 40 sagittal slices were acquired perpendicular to the joint surface. Total acquisition time was 2.25 min.

For perfusion imaging, a dynamic T1-weighted GE turbo FLASH sequence and two T1-weighted GE 3D-FLASH sequences with two different flipangles were acquired; the contrast agent was injected 20 s after sequence initiation. Total acquisition time was 7.20 min. B_1_ shimming was applied to maximize image quality.

The detailed sequence parameters were as follows:

Coronal T1 turbo spin echo (TSE) (TR/TE 862/27 ms; flip angle 150°; slice thickness 2.5 mm; field of view 140 × 140 mm; imaging matrix: 512 × 512; pixel size 0.3 × 0.3 mm), coronal STIR (TR/TE, 5560/31 ms; flip angle 120°; slice thickness 2.5 mm; 8.0; slice thickness 3.0 mm; field of view 140 × 140 mm; imaging matrix: 448 × 312; pixel size 0.3 × 0.3 mm), sagittal proton density (PD) TSE fat-saturated (TR/TE 3150/47 ms, flip angle 150°, slice thickness 2.5 mm, field of view 60 × 150 mm; imaging matrix: 448 × 182; pixel size 0.3 × 0.3 mm), transversal T2 TSE fat-saturated (TR/TE 5693.8/89 ms, flip angle 180°, slice thickness 3.0 mm, field of view 160 × 160 mm; imaging matrix: 512 × 358; pixel size 0.3 × 0.3 mm), transversal T1 SE fat-saturated after intravenous (iv) contrast administration (TR/TE 807/16 ms; flip angle 90°; slice thickness 3.0 mm; field of view 130 × 130 mm; imaging matrix: 384 × 288; pixel size 0.3 × 0.3 mm), coronal T1 TSE after iv contrast (TR/TE 862/27 ms; flip angle 150°; slice thickness 2.5 mm; field of view 140 × 140 mm; imaging matrix: 512 × 512; pixel size 0.3 × 0.3 mm), 3D FLASH GE (TR/TE 5.8/1.9 ms; flip angle 5/26°; slice thickness 3.0 mm; field of view 65 × 110 mm; imaging matrix: 384 × 228; pixel size 0.3 × 0.3 mm) and T1 GE Turbo FLASH (TR/TE 5.8 / 1.9 ms; flip angle 5°; slice thickness 3.0 mm; field of view 140 × 140 mm; imaging matrix: 128 × 96; pixel size 1.1 × 1.1 mm).

### Image Analysis

MR images were independently read and analyzed by two radiologists (DBA and CS, trained in musculoskeletal imaging with 3 and 8 years experience) and one rheumatologist (PS, trained in musculoskeletal imaging with 8 years of experience) according to the OMCERACT PsAMRIS guidelines (9). In addition, joint space width (JSW; minimal distance in mm between the proximal and distal bone surface) and total cartilage thickness (TCT; sum of the proximal and distal cartilage layer) were measured for each MCP, PIP and DIP joint of finger 2–5. Measurements were performed perpendicular to the subchondral bone in the medial part of the joint using the inbuilt digital caliper tool of the picture archiving and communication system (PACS, Sectra Workstation IDS7, Sectra AB, Linköping, Sweden) on sagittal PDw images.

Perfusion in the MCP, PIP, and DIP joints of finger 2-5 was evaluated with quantitative and semi-quantitative analysis using The DCE Tool (The DCE Tool for ClearCanvas 2.0 SP1, http://thedcetool.com) as described in previously published studies of our institute (10). The quantitative analysis of this tool is based upon the Tofts model (22). Perfusion analysis requires the knowledge of T1 relaxation times. Therefore, the T1w GE 3D FLASH sequence with variable flip angles was used for a pixel-based calculation of the T1 time. For this calculation we applied the following formula:

$$\begin{array}{l} T1\left(x,\;y,\;z\right)=\frac{\text{TR}}{\ln\left[\frac{\sin\left(\alpha 1\right)\cos\left(\alpha 2\right)-Q\left(x,\;y,\;z\right)\sin\left(\alpha 2\right)\cos\left(\alpha 1\right)}{\sin\left(\alpha 1\right)-Q\;\left(x,\;y,\;z\right)\sin\left(\alpha 2\right)}\right)} \end{array}$$

where

$$\begin{array}{l} Q\;\left(x,\;y,\;z\right)=\frac{S\alpha 1\;\left(x,\;y,z\right)}{S\alpha 2\;\left(x,\;y,z\right)} \end{array}$$

And S_α1_ (x, y, z) and S_α2_ (x, y, z) are the corresponding pixel intensities to flip angles α_1_ and α_2_. Then, the T1 relaxation was used for the perfusion analysis.

A region of interest (ROI) was placed on the radial and ulnar side of each joint by one reader (DBA). After ROI placement a second reader (CS) confirmed the optimal placement before each measured signal intensity was used to determine a corresponding concentration time curve using the following formula:

$$\begin{array}{l} C_{GD}\;\left(t\right)=\frac{\text{S }\left(t\right)-S_{0}}{S_{0}T_{10}R} \end{array}$$

where T_10_ is the native T1 time, R = 4.5 s^−1^ mM^−1^ is the relaxivity of the contrast agent, S is the average signal intensity in the ROI and S_0_ is the average signal intensity in the ROI in absence of the contrast agent. This Tofts model requires the knowledge of the arterial input function (AIF). AIF can be calculated individually from the blood signal or, alternatively, a population average can be used (22). In this study, we used an analytically described AIF population average that can be used at any temporal resolution (22).

The following perfusions parameters were calculated:

Perfusion parameters are displayed and explained in Table 1: K_Trans_, k_ep_ (quantitative parameters) and IAUC (integral of the signal curve over time), initial slope and peak (semiquantitative parameters).
For compositional analyses of cartilage quality with dGEMRIC, motion correction was applied using STROKETOOL (Digital Image Solutions, Frechen, Germany, http://www.digitalimagesolutions.de) for all images to reduce movement artifacts. This tool has been validated for dGEMRIC analyses of the finger joints and corrects for patient motion between the measurements using a dedicated image registration method (23).
Readers were allowed to adjust the window settings as required to guarantee optimal visualization of the intra- and periarticular structures for ROI placement. T1 maps were analyzed by first defining regions-of-interest (ROIs) on the central sagittal slice. ROI outlines comprising the full thickness of the proximal and distal portion of the articular cartilage of MPC, PIP and DIP joints of finger 2-5 were manually defined on the morphological images of the 3D T1-weighted FLASH sequence with the flip angle of 5° for dGEMRIC. Particular care was taken to exclude artifacts and surrounding structures such as synovial fluid and cortical bone. Consequently, four ROIs were set per digit (i.e., metacarpal, base of proximal phalanx, apex of proximal phalanx, and base of intermediate phalanx) and 16 ROIs per patient (i.e., four ROIs of four digits) and visually checked by the second and third reader to confirm that only cartilage was included. Next, ROIs were copied to the corresponding slices of the color-coded T1 parameter maps. Further analyses involved the pixel-wise calculation post-contrast T1 values as before (17, 24, 25). More specifically, the T1 maps representing the spatially resolved dGEMRIC indices were analyzed in terms of the ROIs as defined above the mean dGEMRIC indices [ms] were recorded. All images were analyzed by two readers (DBA and CS, radiologists) who were blinded for patients' data.

**Table 1:** Description of quantitative and semi-quantitative perfusion parameters. IAUC: initial area under the curve.

**Quantitative parameters**		**Semi-quantitative parameters**		
**K_Trans_**	**k_ep_**	**IAUC**	**Initial slope**	**Peak**
Transfer constant between EES and blood plasma	KTrans/Ve, V_e_: relative volume of EES	Integral of the signal curve over time starting at the onset time (t_onset_) of the bolus	Slope of the signal curve determined by linear regression within the initial seconds after onset	Maximal signal enhancement

*EES, extravascular extracellular space*.

### Statistical Analysis

All statistical analyses were performed using SPSS software (IBM, version 22, Armonk, NY, USA). For descriptive analysis mean, standard deviation, minimum, and maximum were calculated. Due to the small sample size and the heterogeneity of our data, non-normal distribution was assumed. For comparison of means, Kruskal-Wallis test and a *post-hoc* Bonferroni test were performed. Correlation analysis was performed between each dGEMRIC indices, total PsAMRIS and all its sub-scores and TCT using the Kendall–Tau correlation coefficient. *p*-values < 0.05 were considered to be significant.

## Results

### Descriptive Analysis of dGEMRIC Indices, Perfusion Parameters, JSW, and TCT at MCP and PIP Joints

The descriptive analysis (mean, standard deviation, and range) of dGEMRIC values, quantitative (K_Trans_, K_ep_) and semi-quantitative (IAUC, initial slope, peak) perfusion parameters, JSW, and TCT of MCP, PIP, and DIP joints and overall are displayed in Table 2.

**Table 2:** Descriptive analysis (mean, standard deviation (SD) and range (maximum, minimum) of quantitative and semi-quantitative perfusion parameters, delayed Gadolinium Enhanced Magnetic Resonance Imaging of Cartilage (dGEMRIC) indices, joint space width (JSW), and total cartilage thickness (TCT) of finger 2–5 at the metacarpophalangeal (MCP), proximal interphalangeal (PIP), and distal interphalangeal (DIP) joint region and overall.

		**K trans ml/g per min**	**K ep 1/min**	**IAUC mM/l per s**	**Initial slope mM/l per s**	**Peak mM/l per s**	**dGEMRIC in ms**	**TCT in mm**	**JSW in mm**
MCP	Mean	0.06	0.18	3.08	0.0023	0.15	542.65	1.15	1.5
	SD	0.04	0.13	2.46	0.002	0.10	130.34	0.26	0.17
	Max	0.14	0.53	8.45	0.007	0.36	828.03	1.59	1.83
	Min	0.02	0.03	0.81	0.0004	0.05	340.4	0.73	1.27
PIP	Mean	0.05	0.17	2.90	0.002	0.15	411.92	0.71	1.02
	SD	0.03	0.13	2.01	0.002	0.08	104.46	0.18	0.24
	Max	0.12	0.65	7.59	0.006	0.31	639.6	1.11	1.49
	Min	0.008	0.04	0.58	0.0004	0.04	237.18	0.38	0.69
DIP	Mean	0.06	0.21	3.72	0.003	0.16	352.86	0.57	0.8
	SD	0.04	0.15	2.72	0.002	0.08	98.75	0.2	0.18
	Max	0.17	0.68	9.96	0.009	0.29	585.03	0.79	1.19
	Min	0.01	0.06	0.43	0.0003	0.04	184.35	0	0.55
Overall	Mean	0.06	277.32	3.11	0.003	0.15	436.30	0.77	1.07
	SD	0.03	802.71	1.88	0.002	0.07	110.09	0.2	0.18
	Max	0.12	3141.11	7.67	0.006	0.30	670.98	1.13	1.44
	Min	0.01	0.04	0.57	0.0004	0.05	253.98	0.40	0.75
	MCP vs PIP	0.359	0.591	0.864	0.531	1.00	**0.019**	**0.029**	**<0.001**
*p*-value	MCP vs DIP	0.724	0.803	0.558	0.818	0.848	**0.001**	**0.02**	**0.007**
	PIP vs DIP	0.079	0.918	0.874	0.896	0.848	0.491	0.566	0.116

*Mean values of each parameter were compared with a Kruskal-Wallis test and a post-hoc Bonferroni test. P-values <0.05 were considered significant and are given in bold type*.

**Figure 1:**
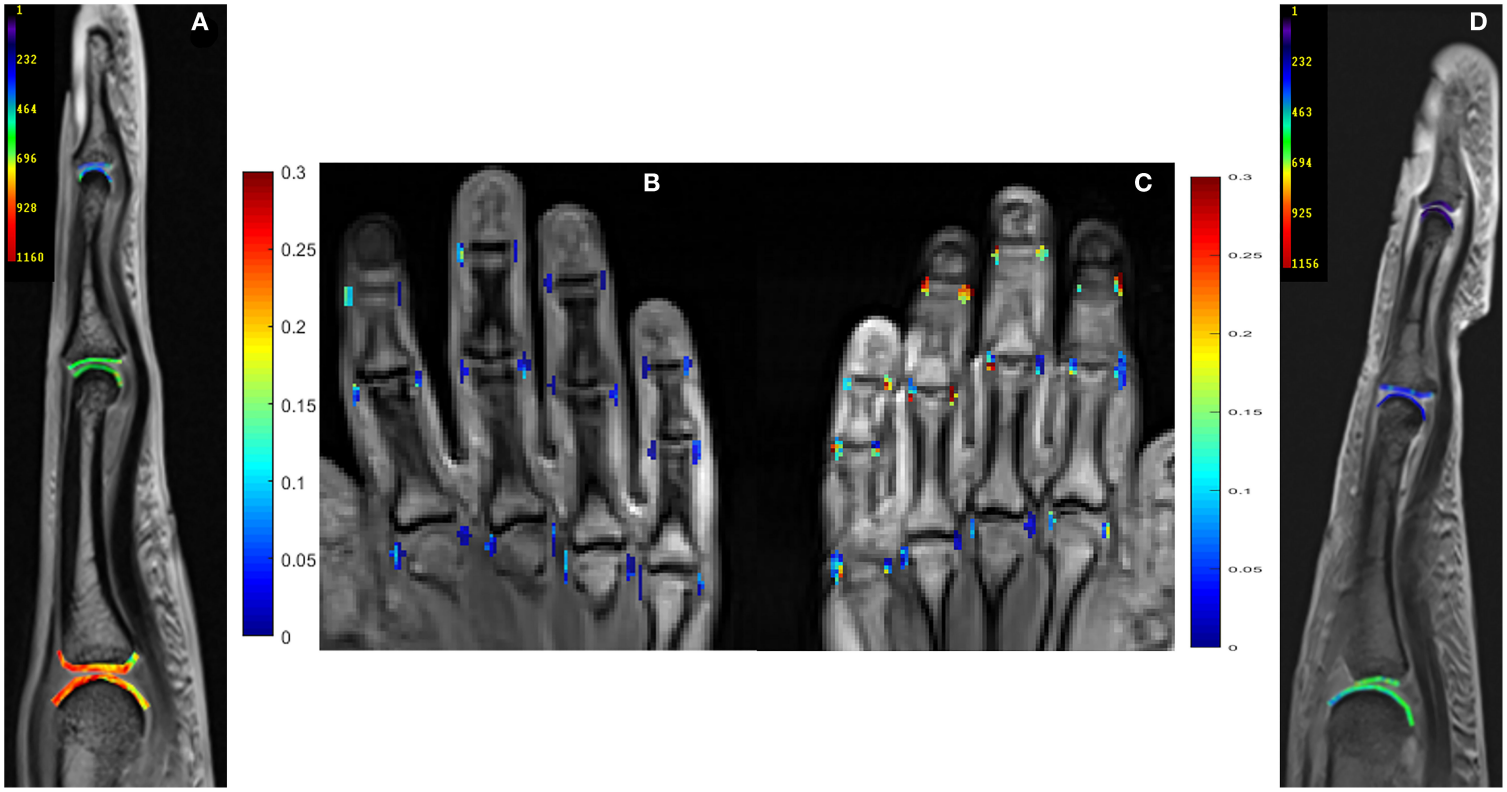
Delayed Gadolinium Enhanced Magnetic Resonance Imaging of Cartilage (dGEMRIC) maps (ms, third digit) and perfusion maps (peak parameter) of metacarpophalangeal (MCP), proximal interphalangeal (PIP), and distal interphalangeal (DIP) joints in 26-year-old male **(A,B)** and a 59-year-old female **(C,D)** with PsA. Lower dGEMRIC values are illustrated in **(D)**, indicating more proteoglycan loss than in **(A)**. Higher peak values are depicted in **(C)**, indicating a higher severity of synovitis than in **(B)**. Peak parameter is illustrated in mM/l per second, dGEMRIC indices in ms.

Perfusion and dGEMRIC maps are shown in Figure 1.

### Correlation Between Perfusion Parameters and JSW, TCT, Total PsAMRIS, Synovitis Sub-score, dGEMRIC Indices, CRP-Levels, and DAS 28

The correlation between perfusion parameters and JSW, TCT, total PsAMRIS, synovitis sub-score, DAS 28, and dGEMRIC indices is illustrated in Table 3.

**Table 3:** Kendall Tau correlation τ between quantitative and semi-quantitative perfusion parameters and dGEMRIC indices, total Psoriatic arthritis magnetic resonance imaging score (PsAMRIS), PsAMRIS sub-score “synovitis.”

		**K_Trans_**		**K_ep_**		**IAUC**		**Initial slope**		**Peak**	
		**τ**	***p***	**τ**	***p***	**τ**	***p***	**τ**	***p***	**τ**	***p***
Overall	JSW	0.1	0.35	−0.1	0.33	0.05	0.64	0.02	0.84	0.04	0.69
	TCT	0.03	0.8	−0.12	0.26	0	0.97	−0.12	0.91	−0.12	0.91
	dGEMRIC	−0.27	**0.014**	−0.29	**0.008**	−0.29	**0.008**	0.32	**0.004**	−0.26	**0.02**
	PsAMRIS	−0.44	0.826	−0.27	0.188	0.09	0.661	0.13	0.51	0.18	0.38
	Synovitis	0.17	0.409	−0.12	0.545	0.26	0.205	0.26	0.205	0.35	0.088
	DAS 28	0.19	0.335	−0.04	0.854	0.27	0.169	0.014	0.952	0.32	0.108
MCP	JSW	0.2	0.3	−0.01	0.96	0.03	0.87	0.03	0.87	0.12	0.55
	TCT	−0.21	0.3	−0.17	0.41	−0.25	0.2	−0.25	0.2	−0.25	0.2
	dGEMRIC	−0.54	**0.01**	−0.02	0.90	−0.51	**0.015**	−0.54	**0.01**	−0.67	**0.002**
	PsAMRIS	0.02	0.912	−0.16	0.44	0.09	0.657	0.14	0.375	0.23	0.268
	Synovitis	0.11	0.612	−0.04	0.866	0.16	0.463	0.23	0.284	0.3	0.159
	DAS28	0.24	0.459	−0.03	0.939	0.22	0.5	0.28	0.385	0.22	0.489
PIP	JSW	0.07	0.73	−0.03	0.88	0.11	0.59	0.17	0.41	0.17	0.41
	TCT	0.12	0.55	−0.06	0.77	0.15	0.43	0.22	0.27	0.22	0.27
	dGEMRIC	−0.43	**0.03**	0.07	0.7	−0.39	0.055	−0.51	**0.015**	−0.51	**0.015**
	PsAMRIS	0.34	0.089	0.02	0.920	0.34	0.089	0.26	0.205	0.44	**0.032**
	Synovitis	0.39	0.053	0.14	0.476	0.41	**0.042**	0.31	0.142	0.45	**0.032**
	DAS28	0.18	0.568	0.05	0.886	0.2	0.536	0.12	0.708	0.22	0.489
DIP	JSW	−0.1	0.62	−0.1	0.62	−0.03	0.87	−0.01	0.96	−0.25	0.21
	TC	−0.05	0.78	−0.1	0.62	0.01	0.96	0.03	0.87	−0.17	0.41
	dGEMRIC	−0.26	0.22	0.1	0.63	−0.18	0.39	−0.15	0.46	−0.08	0.71
	PsAMRIS	0.54	**0.007**	0.10	0.621	0.48	**0.018**	0.46	**0.024**	0.43	**0.032**
	Synovitis	0.22	0.294	−0.01	0.956	0.17	0.407	0.15	0.473	0.21	0.294
	DAS28	−0.06	0.848	0.01	0.901	−0.04	0.901	−0.12	0.722	0.07	0.829

*Disease Activity Score 28 (DAS 28), JSW and TCT of finger 2–5 at the MCP, PIP, and DIP joint level and overall. p-values < 0.05 were considered significant and are written in bold type*.

There was no significant correlation between any perfusion parameter and JSW or TCT, neither overall nor at any joint level (MCP, PIP, DIP).

Overall, there was a significant negative correlation between dGEMRIC indices and all perfusion parameters except k_ep_. The strongest correlation was found at the MCP joint level.

No significant correlation was seen between any perfusion parameter and overall PsAMRIS and/or synovitis sub-score at the MCP joints and overall. For PIP joints, we found a significant correlation for the parameter peak and total PsAMRIS (τ = 0.44, *p* = 0.032) and for the parameters IAUC and peak and the synovitis sub-score (τ = 0.41, *p* = 0.042; τ = 0.451, *p* = 0.032). At the DIP level, there was a significant correlation between the perfusions parameters K_Trans_, IAUC, initial slope, and peak and the total PsAMRIS (τ = 0.54, *p* = 0.07; τ = 0.48, *p* = 0.018; τ = 0.46, *p* = 0.024; τ = 0.43, *p* = 0.032). Further, no significant correlations were found between perfusion parameters and DAS 28 as well as serum CRP levels.

**Figure 2:**
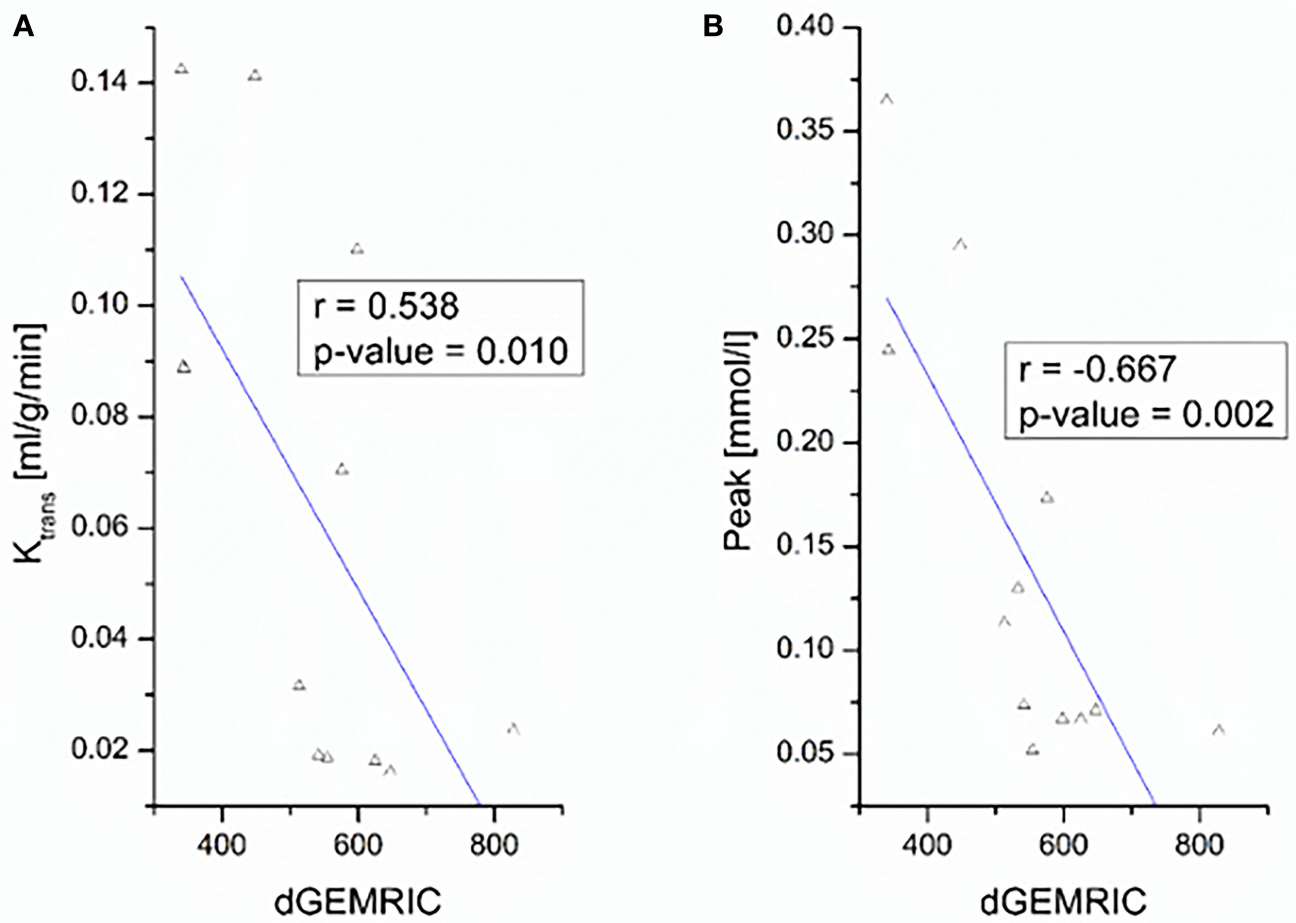
Correlation between dGEMRIC indices and perfusion parameters K_Trans_ **(A)** and peak **(B)** of finger 2–5 at the MCP joint level.

The negative correlations between dGEMRIC values and the quantitative parameter K_Trans_ and the semi-quantitative parameter peak at the MCP joint level are depicted in Figure 2.

## Discussion

Cartilage degradation is a known feature of PsA that can reliably be assessed by dGEMRIC (26). However, as opposed to RA, research is sparse on the role of cartilage in the pathogenesis of PsA. DCE MRI is a valid tool for the evaluation of inflammation in a given joint that has been validated for many types of arthritis (11, 12). In this study, we set out to investigate the relationship between joint inflammation and cartilage loss measured by DCE MRI and dGEMRIC.

We found a significant negative correlation of dGEMRIC indices and quantitative and semi-quantitative perfusion parameters, wherein MCP and PIP joints showed the highest values. The exact reason for the missing correlations at the DIP joints remain unclear, but might be due to a constitutively different proteoglycan content of cartilage along the finger joints or a higher loss of proteoglycans at MCP and PIP than at DIP joints in this specific population of PsA patients. This indicates that molecular cartilage loss is associated with inflammatory joint changes in patients with established PsA, and hence, high inflammation of joints leads to cartilage damage. These findings concur with previous research on cartilage loss, synovial inflammation and perfusion parameters in patients with early RA (10, 16, 20). Since biochemical MRI detects molecular cartilage degradation preceding structural damage, it might be applicable as a monitoring tool for very early disease-related joint changes in PsA.

The association of perfusion parameters and PsAMRIS (sub-scores) has not yet been evaluated. Previous studies on RA showed that perfusion parameters highly correlated with RAMRIS and histological synovitis in affected patients (10, 13, 27–29). As opposed to these findings, we found heterogeneous correlations of perfusion parameters and total PsAMRIS, as well as the synovitis sub-score in PsA patients. DCE MRI is known to indicate the severity of inflammation at a given joint; that is why one could have expected a strong association between perfusion parameters and PsAMRIS. However, previous research using DCE MRI has partially shown that PsA and RA can differ regarding the degree of their synovial enhancement, despite indistinguishable appearances on non-dynamic MRI (30, 31). Also, the synovial involvement of PsA histologically differs from RA regarding the extent of inflammation, synovial thickness, and blood supply (31–33). These differences of synovial changes are potentially due to the different pathogenesis of both entities, with RA being primarily a synovial and PsA being an entheseal-driven disease (34, 35). Therefore, the visual degree of synovitis using PsAMRIS could be over- or underrepresenting synovitis measured by DCE MRI, possibly due to a disease-specific type of synovial involvement. Further, for PsAMRIS scoring, we used coronal and transversal planes, wherein for DCE MRI, we only considered radial and ulnar ROI in coronal slices, which could also contribute to heterogeneous correlations of perfusion parameters and synovitis sub-scores. Additionally, the heterogeneity between MCP, PIP, and DIP joints could be explained by the known circumstances that the state of diffusion equilibrium is reached faster in smaller compared to larger joints (36).

Further, no significant correlations were found between perfusion parameters and clinical disease activity as measured by DAS 28. Previous studies have shown that MRI is more sensitive than clinical scores at the detection of joint inflammation (37, 38). Some studies even demonstrated an radiological progression despite clinical remission and postulated a “silent progression” (39–41). That is why, the lacking correlation of imaging features and clinical data could be due to the superior sensitivity of MRI, especially since a high-field MRI scanner and a dedicated hand-coil have been used resulting in high-resolution imaging.

### Our Study Has Limitations

Firstly, our study population of PsA patients had a small sample size. That is why our results should only be considered exploratory and need confirmation by further research with larger populations.

Secondly, we did not use a synovial and cartilage biopsy as a means of validation regarding the extent of synovitis and the cartilage composition. However, previous studies have already histologically validated both DCE MRI and dGEMRIC data (13, 42).

In conclusion, there is a potential association between early cartilage loss and acute synovial inflammation in small finger joints of PsA patients.

## Data Availability Statement

The datasets generated for this study are available on request to the corresponding author.

## Ethics Statement

The studies involving human participants were reviewed and approved by Ethics committee of the Heinrich-Heine University. The patients/participants provided their written informed consent to participate in this study.

## Author Contributions

DA: acquisition and analysis and interpretation of data. Draft and design of the work. PS: design and conception of the study. Analysis and interpretation of data. Draft and design of the work. Revision of the work. BO, MS, SV, and KR: analysis and interpretation of data. Draft and design of the work. Revision of the work. MF and AM-L: interpretation and analysis of data. Draft and design of the work. Revision of the work. CS: conception and design of the study. Interpretation and analysis of data. Draft and design of the work. Revision of the work. All authors read and approved the final manuscript. All authors contributed to the article and approved the submitted version.

## Conflict of Interest

The authors declare that the research was conducted in the absence of any commercial or financial relationships that could be construed as a potential conflict of interest.

## References

[1] Sewerin P Brinks R Schneider M Haase I Vordenbäumen S. Prevalence and incidence of psoriasis and psoriatic arthritis. Ann Rheum Dis. (2019) 78:286–7. 10.1136/annrheumdis-2018-214065 30242033

[2] Coates LC Navarro-Coy N Brown SR Brown S McParland L Collier H. The TICOPA protocol (TIght COntrol of Psoriatic Arthritis): a randomised controlled trial to compare intensive management versus standard care in early psoriatic arthritis. BMC Musculoskelet Disord. (2013) 14:101. 10.1186/1471-2474-14-101 23517506 PMC3610193

[3] Singh JA Guyatt G Ogdie A Gladman DD Deal C Deodhar A Special article: 2018 American College of Rheumatology/National psoriasis foundation guideline for the treatment of psoriatic arthritis. Arthritis Care Res. (2019) 71:2–29. 10.1002/art.40726 PMC8265826 30499259

[4] Coates LC. Treating to target in psoriatic arthritis. Curr Opin Rheumatol. (2015) 27:107. 10.1097/BOR.0000000000000140 25603035

[5] Coates LC Lubrano E Perrotta FM Emery P Conaghan PG Helliwell PS. What should be the primary target of “treat to target” in psoriatic arthritis? J Rheumatol. (2019) 46:38–42. 10.3899/jrheum.180267 30219765

[6] Tillett W Costa L Jadon D Wallis D Cavill C McHugh J. The ClASsification for Psoriatic ARthritis (CASPAR) criteria–a retrospective feasibility, sensitivity, and specificity study. J Rheumatol. (2012) 39:154–6. 10.3899/jrheum.110845 22089469

[7] Coates LC Conaghan PG D'Agostino MA Wit M de FitzGerald O Kvien TK. Remission in psoriatic arthritis-where are we now? Rheumatology. (2018) 57:1321–31. 10.1093/rheumatology/kex344 29045698

[8] Yue J Wu D Tam L-S. The role of imaging in early diagnosis and prevention of joint damage in inflammatory arthritis. Expert Rev Clin Immunol. (2018) 14:499–511. 10.1080/1744666X.2018.1476849 29754519

[9] Ostergaard M McQueen F Wiell C Bird P Bøyesen P Ejbjerg B. The OMERACT psoriatic arthritis magnetic resonance imaging scoring system (PsAMRIS): definitions of key pathologies, suggested MRI sequences, and preliminary scoring system for PsA Hands. J Rheumatol. (2009) 36:1816–24. 10.3899/jrheum.090352 19671819

[10] Müller-Lutz A Schleich C Sewerin P Gross J Pentang G Wittsack H-J. Comparison of quantitative and semiquantitative dynamic contrast-enhanced MRI with respect to their correlation to delayed gadolinium-enhanced MRI of the cartilage in patients with early rheumatoid arthritis. J Comput Assist Tomogr. (2015) 39:64–9. 10.1097/RCT.0000000000000164 25340588

[11] Schwenzer NF Kötter I Henes JC Schraml C Fritz J Claussen CD. The role of dynamic contrast-enhanced MRI in the differential diagnosis of psoriatic and rheumatoid arthritis. AJR Am J Roentgenol. (2010) 194:715–20. 10.2214/AJR.09.2671 20173150

[12] Cimmino MA Barbieri F Boesen M Paparo F Parodi M Kubassova O. Dynamic contrast-enhanced magnetic resonance imaging of articular and extraarticular synovial structures of the hands in patients with psoriatic arthritis. J Rheumatol Suppl. (2012) 89:44–8. 10.3899/jrheum.120242 22751591

[13] Vordenbäumen S Schleich C Lögters T Sewerin P Bleck E Pauly T. Dynamic contrast-enhanced magnetic resonance imaging of metacarpophalangeal joints reflects histological signs of synovitis in rheumatoid arthritis. Arthritis Res Ther. (2014) 16:452. 10.1186/s13075-014-0452-x 25270553 PMC4201730

[14] Bhattaram P Chandrasekharan U. The joint synovium: A critical determinant of articular cartilage fate in inflammatory joint diseases. Semin Cell Dev Biol. (2017) 62:86–93. 10.1016/j.semcdb.2016.05.009 27212252

[15] Østergaard M Peterfy CG Bird P Gandjbakhch F Glinatsi D Eshed I. The OMERACT rheumatoid arthritis magnetic resonance imaging (MRI) scoring system: updated recommendations by the OMERACT MRI in arthritis working group. J Rheumatol. (2017) 44:1706–12. 10.3899/jrheum.161433 28811353

[16] Herz B Albrecht A Englbrecht M Welsch GH Uder M Renner N. Osteitis and synovitis, but not bone erosion, is associated with proteoglycan loss and microstructure damage in the cartilage of patients with rheumatoid arthritis. Ann Rheum Dis. (2014) 73:1101–6. 10.1136/annrheumdis-2012-202850 23625980

[17] Miese F Buchbender C Scherer A Wittsack H-J Specker C Schneider M. Molecular imaging of cartilage damage of finger joints in early rheumatoid arthritis with delayed gadolinium-enhanced magnetic resonance imaging. Arthritis Rheum. (2012) 64:394–9. 10.1002/art.33352 21952736

[18] van Tiel J Kotek G Reijman M Bos PK Bron EE Klein S. Is T1ρ mapping an alternative to delayed gadolinium-enhanced MR imaging of cartilage in the assessment of sulphated glycosaminoglycan content in human osteoarthritic knees? An *in vivo* validation study. Radiology. (2016) 279:523–31. 10.1148/radiol.2015150693 26588020

[19] Abrar DB Schleich C Brinks R Vordenbäumen S Frenken M Goertz C Is a simplified version of PsAMRIS (sPsAMRIS) a potential tool for therapy monitoring in established psoriatic arthritis? Res Square [Preprint]. (2019). 10.21203/rs.2.14892/v1

[20] Sewerin P Schleich C Brinks R Müller-Lutz A Fichter F Eichner M. Assessing associations of synovial perfusion, cartilage quality, and outcome in rheumatoid arthritis using dynamic contrast-enhanced magnetic resonance imaging. J Rheumatol. (2020) 47:15–9. 10.3899/jrheum.180832 30877219

[21] Sewerin P Schleich C Brinks R Müller-Lutz A Fichter F Eichner M. Synovial perfusion assessed by dynamic contrast-enhanced MRI is associated to treatment response, remission, and cartilage quality in rheumatoid arthritis. J Rheumatol. (2019). 10.3899/jrheum.180832. [Epub ahead of print]. 30877219

[22] Tofts PS T1-weighted DCE imaging concepts: modelling, acquisition and analysis. MAGNETOM Flash. (2010) 2010:30–9.

[23] Miese F Kröpil P Ostendorf B Scherer A Buchbender C Quentin M. Motion correction improves image quality of dGEMRIC in finger joints. Eur J Radiol. (2011) 80:e427–31. 10.1016/j.ejrad.2011.01.006 21353423

[24] Miese FR Ostendorf B Wittsack H-J Reichelt DC Mamisch TC Zilkens C. Metacarpophalangeal joints in rheumatoid arthritis: delayed gadolinium-enhanced MR imaging of cartilage–a feasibility study. Radiology. (2010) 257:441–7. 10.1148/radiol.10100459 20807848

[25] Sewerin P Müller-Lutz A Abrar DB Odendahl S Eichner M Schneider M. Prevention of the progressive biochemical cartilage destruction under methotrexate therapy in early rheumatoid arthritis. Clin Exp Rheumatol. (2019) 37:179–85. 29998824

[26] Sewerin P Schleich C Vordenbäumen S Ostendorf B. Update on imaging in rheumatic diseases: cartilage. Clin Exp Rheumatol. (2018) 36 Suppl 114:139–44. 30296981

[27] Hodgson R Grainger A O'Connor P Barnes T Connolly S Moots R. Dynamic contrast enhanced MRI of bone marrow oedema in rheumatoid arthritis. Ann Rheum Dis. (2008) 67:270–2. 10.1136/ard.2007.077271 17965120

[28] Boesen M Kubassova O Bouert R Axelsen MB Ostergaard M Cimmino MA. Correlation between computer-aided dynamic gadolinium-enhanced MRI assessment of inflammation and semi-quantitative synovitis and bone marrow oedema scores of the wrist in patients with rheumatoid arthritis–a cohort study. Rheumatology. (2012) 51:134–43. 10.1093/rheumatology/ker220 22075065

[29] Wojciechowski W Tabor Z Urbanik A. Assessing synovitis based on dynamic gadolinium-enhanced MRI and EULAR-OMERACT scores of the wrist in patients with rheumatoid arthritis. Clin Exp Rheumatol. (2013) 31:850–6. 24093565

[30] Sudoł-Szopińska I Pracoń G. Diagnostic imaging of psoriatic arthritis. Part II: magnetic resonance imaging and ultrasonography. J Ultrason. (2016) 16:163–74. 10.15557/JoU.2016.0018 27446601 PMC4954862

[31] Narváez J Narváez JA Albert M de Gómez-Vaquero C Nolla JM. Can magnetic resonance imaging of the hand and wrist differentiate between rheumatoid arthritis and psoriatic arthritis in the early stages of the disease? Semin Arthritis Rheum. (2012) 42:234–45. 10.1016/j.semarthrit.2012.03.016 22595641

[32] Sankowski AJ Lebkowska UM Cwikła J Walecka I Walecki J Psoriatic arthritis. Pol J Radiol. (2013) 78:7–17. 10.12659/PJR.883763 PMC3596149 23493653

[33] Sudoł-Szopińska I Płaza M Pracoń G. Selected issues in diagnostic imaging of spondyloarthritides: psoriatic arthritis and juvenile spondyloarthritis. Reumatologia. (2016) 54:310–7. 10.5114/reum.2016.64908 28115782 PMC5241368

[34] McGonagle D Hermann K-GA Tan AL. Differentiation between osteoarthritis and psoriatic arthritis: implications for pathogenesis and treatment in the biologic therapy era. Rheumatology. (2015) 54:29–38. 10.1093/rheumatology/keu328 25231177 PMC4269795

[35] Schett G Coates LC Ash ZR Finzel S Conaghan PG Structural damage in rheumatoid arthritis, psoriatic arthritis, and ankylosing spondylitis: traditional views, novel insights gained from TNF blockade, and concepts for the future. Arthritis Res Ther. (2011) 2011:S4 10.1186/1478-6354-13-S1-S4 PMC3123965 21624183

[36] Peterfy CG. MR imaging. Baillieres Clin Rheumatol. (1996) 10:635–78. 10.1016/S0950-3579(96)80055-0 8958383

[37] Østergaard M Hansen M Stoltenberg M Jensen KE Szkudlarek M Pedersen-Zbinden B. New radiographic bone erosions in the wrists of patients with rheumatoid arthritis are detectable with magnetic resonance imaging a median of two years earlier. Arthritis Rheum. (2003) 48:2128–31. 10.1002/art.11076 12905465

[38] Ejbjerg BJ Vestergaard A Jacobsen S Thomsen HS Østergaard M. The smallest detectable difference and sensitivity to change of magnetic resonance imaging and radiographic scoring of structural joint damage in rheumatoid arthritis finger, wrist, and toe joints: a comparison of the OMERACT rheumatoid arthritis magnetic resonance imaging score applied to different joint combinations and the Sharp/van der Heijde radiographic score. Arthritis Rheum. (2005) 52:2300–6. 10.1002/art.21207 16052593

[39] Møller-Bisgaard S Hørslev-Petersen K Ejbjerg BJ Boesen M Hetland ML Christensen R. Impact of a magnetic resonance imaging-guided treat-to-target strategy on disease activity and progression in patients with rheumatoid arthritis (the IMAGINE-RA trial): study protocol for a randomized controlled trial. Trials. (2015) 16:178. 10.1186/s13063-015-0693-2 25896862 PMC4417239

[40] Sewerin P Vordenbaeumen S Hoyer A Brinks R Buchbender C Miese F. Silent progression in patients with rheumatoid arthritis: is DAS28 remission an insufficient goal in RA? Results from the German Remission-plus cohort. BMC Musculoskelet Disord. (2017) 18:163. 10.1186/s12891-017-1528-y 28420375 PMC5395882

[41] Hetland ML Stengaard-Pedersen K Junker P Østergaard M Ejbjerg BJ Jacobsen S. Radiographic progression and remission rates in early rheumatoid arthritis - MRI bone oedema and anti-CCP predicted radiographic progression in the 5-year extension of the double-blind randomised CIMESTRA trial. Ann Rheum Dis. (2010) 69:1789–95. 10.1136/ard.2009.125534 20444751

[42] Schmaranzer F Arendt L Liechti EF Nuss K Rechenberg B von Kircher PR. Do dGEMRIC and T2 imaging correlate with histologic cartilage degeneration in an experimental ovine FAI model? Clin Orthop Relat Res. (2019) 477:990–1003. 10.1097/CORR.0000000000000593 30507833 PMC6494333

